# Enhancing interacting residue prediction with integrated contact matrix prediction in protein-protein interaction

**DOI:** 10.1186/s13637-016-0051-z

**Published:** 2016-10-22

**Authors:** Tianchuan Du, Li Liao, Cathy H. Wu

**Affiliations:** Department of Computer and Information Sciences, University of Delaware, Newark, DE 19716 USA

**Keywords:** Protein-protein interaction, Contact matrix prediction, Interaction site prediction, Machine learning

## Abstract

Identifying the residues in a protein that are involved in protein-protein interaction and identifying the contact matrix for a pair of interacting proteins are two computational tasks at different levels of an in-depth analysis of protein-protein interaction. Various methods for solving these two problems have been reported in the literature. However, the interacting residue prediction and contact matrix prediction were handled by and large independently in those existing methods, though intuitively good prediction of interacting residues will help with predicting the contact matrix. In this work, we developed a novel protein interacting residue prediction system, contact matrix-interaction profile hidden Markov model (CM-ipHMM), with the integration of contact matrix prediction and the ipHMM interaction residue prediction. We propose to leverage what is learned from the contact matrix prediction and utilize the predicted contact matrix as “feedback” to enhance the interaction residue prediction. The CM-ipHMM model showed significant improvement over the previous method that uses the ipHMM for predicting interaction residues only. It indicates that the downstream contact matrix prediction could help the interaction site prediction.

## Introduction

Protein-protein interactions (PPIs) play crucial roles in many biological processes in living organisms, such as immune response, enzyme catalysis, and signal transduction. Acquiring knowledge of the interfacial regions between interacting proteins is not only helpful in understanding protein functions and elucidating signal transduction networks but also critical for structure-based drug design and disease treatment [1, 2]. The identification of the protein-protein interaction sites (PPISs) or the PPI interacting residues holds great therapeutic potential for the rational design of molecules modulating and mimicking their effects [1–3]. Previous research on PPI site prediction and analysis has been summarized in some recent reviews [3–10].

While just knowing the interaction site is good enough for many applications, we further want to know how those interacting residues across the interface between two interacting proteins are paired up because the residue-residue contact information of two interacting proteins can provide further insights into interactions and specific target candidates for mutagenesis. Computational methods that can predict the detailed residue-residue contact information from pure protein sequences have been reported [11, 12]. The detailed residue-residue contact information of an interacting sequence pair can be viewed as a contact matrix with rows and columns corresponding to the residues in the two interacting sequences respectively, and the element of the matrix indicates whether the corresponding pair of residues interact or not. The contact matrix is a binary-valued matrix, in which 1 implies that the two corresponding residues are in contact and 0 implies that the two corresponding residues are not in contact. Fisher scores extracted from ipHMMs of interacting domains were used with support vector machine (SVMs) to predict the contact points in previous research [11]. In Ovchinnikov et al., covariance between residues across the interface is used to predict the residue contact by assigning a so-called GREMLIN score for each residue pairs.

However, the task of predicting interacting residues and the task of predicting contact matrix were handled pretty much separately and independently in literature. Indeed, these two tasks are at the two different levels of the in-depth analysis of protein-protein interaction. Intuitively, better interacting residue prediction can help with contact matrix prediction, by reducing the search space. But, can we also use the contact matrix prediction to help with interacting prediction in return? Apparently, a contact matrix for two interacting proteins carries more detailed information about the interaction than just knowing interacting residues in individual proteins. In other words, if we have a correct contact matrix, we automatically know where the interacting residues are in the two proteins, whereas the opposite is not true. However, in reality, the ground-truth contact matrix is not known; what is available is a contact matrix predicted from sequence and/or structural information, which may contain false positives, or false negatives, or both. Still. It is intriguing to ask: can we leverage some useful information from the prediction contact matrix to enhance the interacting residue prediction? It has been shown that the matrix-like features can be used for protein interaction site prediction, for example, the probability density maps (PDM) describing likelihoods of contacts [4, 13]. Note that a PDM is within one protein sequence at atomic level which carries different information from the contact matrix. The contact matrix between two interacting proteins is at the residue level.

In this work, we developed a novel machine learning approach (contact matrix-interaction profile hidden Markov model (CM-ipHMM)) to predicting interacting residues with the integration of predicted contact matrix prediction for better accuracy. In doing so, we expect to leverage what is learned from contact matrix prediction and utilize the predicted contact matrix as “feedback” to enhance the interaction site prediction. We formulate the interacting residue (PPIR) prediction problem in the following way. Given the sequences of two interacting proteins, we first make a PPIR prediction with the ipHMM model. Then, we make a contact matrix prediction with an SVM model. Finally, we integrate the PPIR prediction and the contact matrix prediction as an input to the CM-ipHMM system for PPIR prediction.

## Method

### Dataset

One effective approach to studying protein-protein interactions is through domain-domain interactions (DDI) [14–17]. Each protein can be characterized by either one domain or a combination of multiple domains. PPI typically involve binding between domains, the basic units of protein folding, evolution, and function. Proteins interact with one another through their specific domains. Therefore, DDIs present an overall view of the protein-protein interaction network within a cell responsible for carrying out various biological and cellular functions [14]. Therefore, the PPI data used for training and testing our machine learning models are studied at DDI level. Several research groups have published their work in organizing and standardizing the existing and known domain-domain interactions [17–20]. The 3DID database is among the most successful and widely used ones, which contains interactions inferred from protein structures in known PDB entries [20, 21].

To serve as a baseline for comparison, we first select the same dataset for the ipHMM PPI interacting residue prediction as reported in the literature [22]. The set of 146 DDI families, each with just one topology interface type, was chosen to build ipHMMs on DDI family level. Each selected DDI family has 10–20 examples and has distinct domains (i.e., the complex formed by two interacting proteins is a heterodimer). Because the time complexity for manipulating contact matrix is O(n^2^), we will have very long processing time for long protein sequences. We further limit the sum of the domain length to be less than 150 residues so that we will have an upper bound of our matrix size, 150^2^, to prevent prohibitive data processing time. The criteria result in 72 DDI families, which are used for training and testing purposes.

### The interaction profile hidden Markov model for interaction site prediction

The ipHMM site prediction was first performed for each sequence pair with the approach as described in [22]. Here, we will give a brief overview. The ipHMM was first developed for the prediction of protein-ligand interaction sites [23]. In [24], Fisher scores are extracted from ipHMM and are used to train a support vector machine to predict domain-domain interaction.

**Fig. 1:**
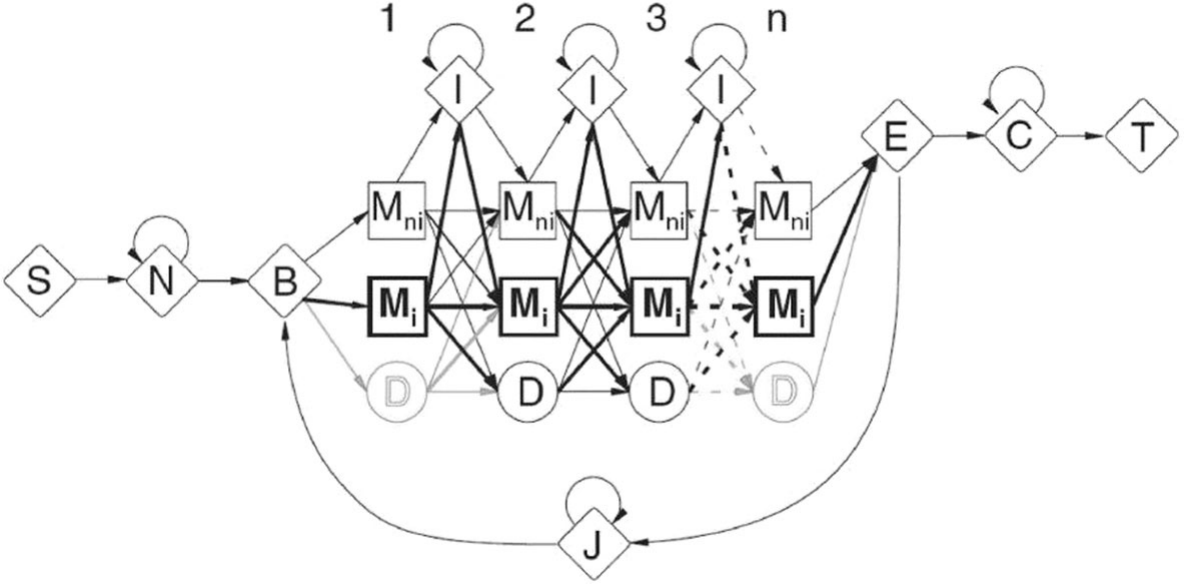
The architecture of the interaction profile hidden Markov model. The match states of the classical pHMM are split into non-interacting (*M*_*ni*_) and interacting (*M*_*i*_) match states. Image credit for Friedrich et al. [23]

Each ipHMM, like pHMMs, is a probabilistic representation of a protein domain family. The ipHMM architecture takes into account both structural information and sequence data. The architecture of the ipHMM follows the same restrictions and connectivity of the HMMER architecture [25]. However, the ipHMM split a match state of the classical pHMM into two states: a non-interacting match (M_ni_) and an interacting match state (M_i_) (Fig. 1). The new match states have the same properties of a match state in the ordinary pHMM, i.e., these interacting match states can emit all amino acid symbols with probabilities. The emission probabilities and transition probabilities are model parameters to be fixed according to the training examples.

The ipHMM is built for each domain family based on the multiple sequence alignment of the member proteins. In the multiple sequence alignment, each match-state residue is labeled with interacting or non-interacting. The transition probability and the emission probability of the ipHMM are estimated using maximum likelihood method based on a multiple sequence alignment of the member proteins in the domain family. Because the statistics for certain states may be extremely low due to the lack of occurrence of the examples, the pseudo count is employed to smooth the possibility. We add one count for each amino acid to every state before we do the counting. For example, if amino acid, lysine, could not be found for a given state, we still count one lysine for that state to avoid the emission possibility of lysine to be zero. Posterior decoding is adopted to predict interacting residues and path-dependent probabilities for every hidden state for ipHMM. After the interacting residues of protein sequence had been predicted, it was compared to the ground-truth data to evaluate the prediction performance.

For each DDI family, we applied the ipHMM site prediction, with leave-one-out cross-validation, for each sequence pair. It was used as a baseline to compare with our CM-ipHMM model. The predicted interacting residues in each sequence were reserved to build an integrated model as described in the following sections.

### The contact matrix prediction model

**Fig. 2:**
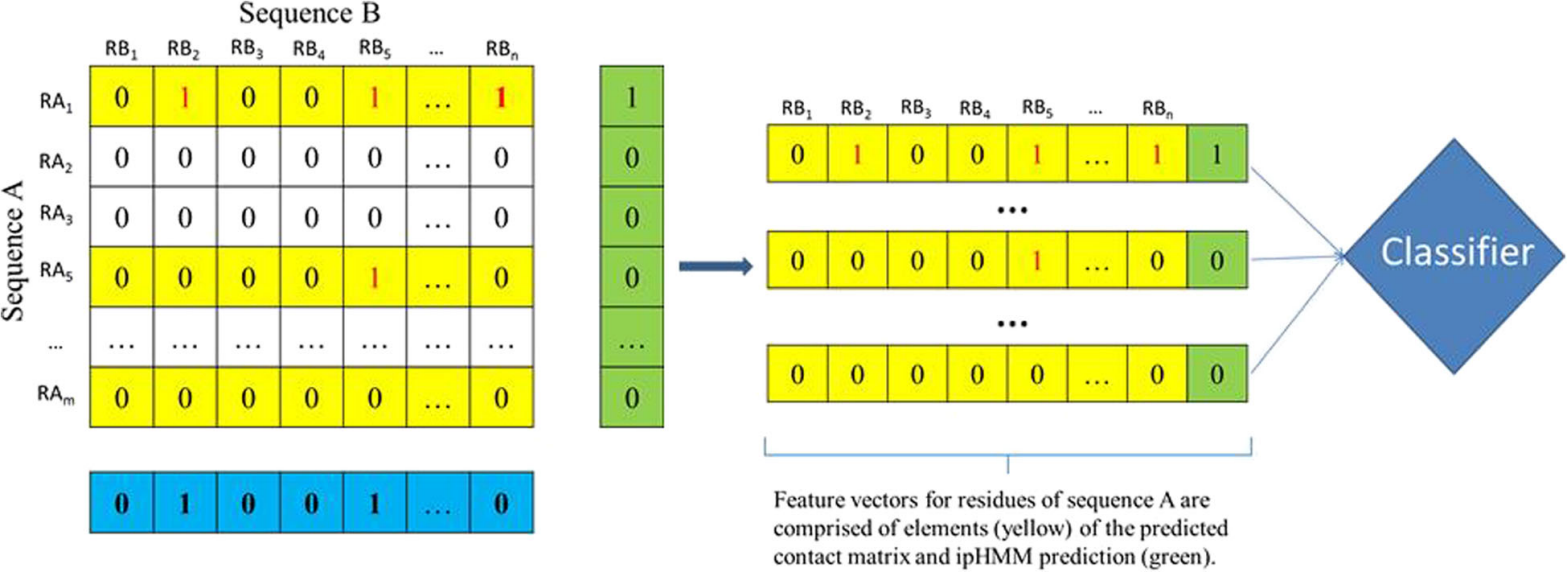
Integrated machine learning classifier with contact matrix prediction and ipHMM prediction. The *green column* and the *blue row* are the ipHMM site prediction result for sequence A and sequence B. The binary matrix is the predicted contact matrix between sequence A and sequence B

For each sequence pair in a given DDI family, we further predict the contact matrix, as shown in the left panel of Fig. 2. In previous research [11], Fisher scores extracted from ipHMMs of interacting domains were used with SVMs to predict the contact points. That method is used for our contact matrix prediction with slight modification. The following is a brief explanation of the method. Firstly, protein domains are identified and profiled using ipHMMs [23, 26]. Secondly, each residue in a domain conserved sequence is represented as a 20-dimensional vector of Fisher scores derived from the ipHMM; these Fisher scores essentially measure how the likelihood of the sequence matching the ipHMM is affected if the residue is mutated to 1 of the 20 possible amino acids. A residue pair in the contact matrix is then represented by a 40-dimensional vector by concatenating the two 20-dimensional vectors. An SVM is trained on residue pairs with known labels (1 for contact and 0 for non-contact) and is then used for predicting whether a given pair of residues forms a contact point or not.

In this work, we extend the feature vectors by including more information. Features collected from different sources can provide various aspects of information to machine learning models for making decisions. Some physiochemical properties of amino acids were demonstrated to be helpful to differentiate interfacial and non-interfacial residues (Wang, [27]). Thus, amino acid index features were combined with the Fisher features to make contact matrix prediction. The amino acid properties for each residue are generated from AAindex database, which is a database of numerical indices representing various physicochemical and biochemical properties of amino acids derived from published literature [28]; 17 features based on amino acid physiochemical properties were used for in this research for as shown in Table 1.

**Table 1:** The selected amino acid properties from AAindex database

Property id	Property description
ANDN920101	Alpha-CH chemical shifts
ARGP820101	Hydrophobicity index
BEGF750101	Conformational parameter of inner helix
BUNA790103	Spin-spin coupling constants 3Jhalpha-NH
BHAR880101	Average flexibility indices
BURA740102	Normalized frequency of extended structure
GEOR030101	Linker propensity from all dataset
CHOP780204	Normalized frequency of N-terminal helix
CHOP780215	Frequency of the 4th residue in turn
JOND920102	Relative mutability
KHAG800101	The Kerr-constant increments
FAUJ880104	STERIMOL length of the side chain
PALJ810107	Normalized frequency of alpha-helix in all-alpha class
RACS820114	Value of theta(i-1)
WERD780103	Free energy change of alpha(Ri) to alpha(Rh)
YUTK870102	Unfolding Gibbs energy in water pH9.0
CHAM830102	A parameter defined from the residuals obtained from the best correlation of the Chou-Fasman parameter of beta-sheet

To capture the association among neighboring residues, a sliding window around the residue of interest is used. Protein interface is formed by some residues that closed to each other in spatial position. A window of 11 residues, centered on the target residue, including the five spatially neighboring residues on each side, was used. As a result, the amino acid index feature of each residue is represented by a vector of dimension, 11 × 17 = 187.

We combine Fisher score features and amino acid index features by concatenating those features for a residue pair of interest. As a result, we will get a feature vector of dimension (20 + 11 × 17) × 2, for each residue pair from the combination of Fisher score features and AA index features. All the features are normalized to be in the range [0,1]. Finally, the features were used to train an SVM model for classification. The SVM model (with an RBF kernel, K(*x*, *y*) = exp (−*γ*|| *x* − *y*))^2^ ) was implemented in Python using Scikit-learn v0.15.2 [29] with default value of the parameters *γ* and *C*, which specifies how much a misclassification be penalized during training in order not to overfit the data. While default values being used in this work, optimizing these parameters, e.g., using grid search, can potentially further improve the performance. This contract matrix prediction model was also trained and tested using leave-one-out fashion as well. The predicted contact matrix for each sequence pair was reserved to build an integrated model described in next section.

### An integrated machine learning model with contact matrix prediction and ipHMM interaction site prediction (CM-ipHMM)

How to make use of the contact matrix prediction information is the key step for the interaction site prediction. We take a row (or a column) of the predicted contact matrix as features for a corresponding residue to feed to the CM-ipHMM machine learning model (Fig. 2). Once we get the ipHMM site prediction for two sequences as shown in Fig. 2 (left, the blue row and the green column), each element in the green column represents the ipHMM site prediction result for a residue on sequence A, and each element in the blue row represents the ipHMM site prediction result for a residue on sequence B. The contact matrix prediction for sequence A and sequence B is shown in Fig. 2 (left). Then, for each residue, we build a feature vector with the corresponding row/column in contact matrix prediction and the residue’s site prediction result with ipHMM to feed into our CM-ipHMM model. For example, each residue in sequence A can be represented by concatenating a corresponding row (yellow) in the predicted contact matrix and a corresponding element in the ipHMM site prediction (green). Then, we use the feature vectors and ground-truth label (interacting residue or not) to train the CM-ipHMM model classifier to predict the interaction site (Fig. 2, right).

In this work, we used logistic regression model as the classifier because of its simplicity and its ability to assign the weights for each feature, which makes easy to interpret the model. The logistic regression model is defined in (1).

1 $$ \Pr \left(Y=1\Big|{X}_1,\dots, {X}_k\right)=\frac{1}{1\kern0.5em +\kern0.5em  \exp \left[-\left({\beta}_0\kern0.5em +\kern0.5em {\beta}_1{X}_1\kern0.5em +\kern0.5em {\beta}_2{X}_2\kern0.5em +\kern0.5em \dots \kern0.5em +\kern0.5em {\beta}_k{X}_k\right)\right]} $$

where Pr(*Y* = 1|*X*_1_,…,*X*_*k*_) estimates the probability of the binary classification class to be 1 given the input features (*X*_1_,…,*X*_*k*_). As shown Fig. 2 (right panel), each residue of sequence A is represented as a feature vector of *n*-dimension, therefore, *k* in Eq. (1) is set to be equal to *n*; and *k* is set to be equal to *m* for sequence B correspondingly. So we build two separate logistic regression models for domain **A** and domain **B**, respectively; each model is trained on member sequences in the domain family and then is tested to make interaction site predictions for the reserved test sequence. The logistic regression model is implemented using a Python package, Scikit-learn v0.15.2 [29], with the default the parameters (penalty = “l2”, *C* = 1, etc.).

### Model training and performance evaluation

For the model training and performance evaluation, we used leave-one-out cross-validation for each DDI family since we normally have limited training sequence pairs. That is, for each sequence pair in a DDI family, we build a model based on other sequence pairs and test on the current sequence pair. Then, we report on the average of results for all the sequences across all the DDI families. During the training process, we randomly oversampled the positive example (interacting residues) to the same number of negative examples to reduce the bias introduced by the imbalanced dataset. To show the effectiveness of using contact matrices and the efficiency of the machine learning model, we also built models with ground-truth contact matrices. The only difference is that we replaced the predicted contact matrix with ground-truth contact matrix in Fig. 2.

We used some commonly used measurements to report the performance, which includes accuracy, precision, recall, F1, and MCC, defined as follows:

$$ \begin{array}{l} Recall=\frac{TP}{TP+FN}\\ {} Precision=\frac{TP}{TP+FP}\\ {} Accuracy=\frac{TP+TN}{TP+TN+FN+FP}\\ {}M\mathrm{C}\mathrm{C}=\frac{TP\times TN\hbox{-} FP\times FN}{\surd \left(TP+FP\right)\left(TP+FN\right)\left(TN+FP\right)\left(TN+FN\right)}\end{array} $$

where TP stands for true positive when a site is correctly predicted as interaction site, TN for true negative when a site is correctly predicted as non-interaction site, FP for false positive when a site is incorrectly predicted as an interaction site, and FN for false negative when a site is incorrectly predicted as non-interaction site.

The prediction performance was evaluated using standard metrics including accuracy, recall, and precision. We applied tenfold cross validation to evaluate the prediction performance for each DDI family. Then, the average performance of tenfold cross-validation was reported over the 123 DDI families. In order to test the statistical significance of improvement with contact matrix information, we performed a paired *t* test between the ipHMM site prediction and the integrated CM-ipHMM model site prediction.

## Results and discussion

**Fig. 3:**
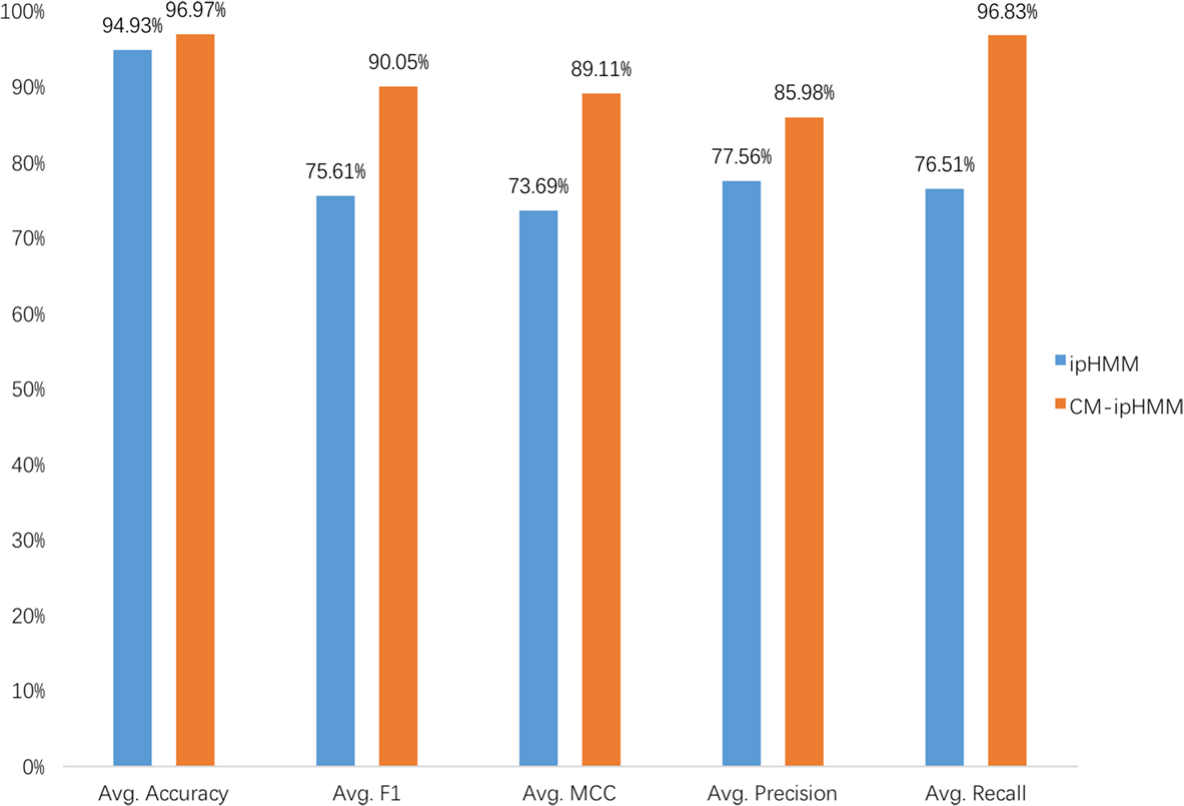
Interaction site prediction performance comparison between the integrated CM-ipHMM model and the ipHMM model. CM-ipHMM (*red*) is the logistic regression model built with the integration of contact matrix prediction and ipHMM interaction site prediction; ipHMM (*blue*) is the interaction profile hidden Markov model used to prediction interaction site

Table 2 shows the interaction site prediction performance of different models. The ipHMM model is based on the method described in Section 2.2, which serves as a baseline. It has the average precision, recall, and MCC as 77.56, 76.51, and 73.69 % respectively, which is similar to what was reported in the literature [22]. Our integrated model, CM-ipHMM, showed significant improvement over the ipHMM interaction site prediction with precision, recall, and MCC as 85.98, 96.83, and 89.11 % respectively. Figure 3 shows that the CM-ipHMM model (red) is constantly better than the ipHMM interaction model (blue) with accuracy, F1, MCC, precision, and recall. Because normally interacting residues are significantly less than non-interacting residues in protein sequences, which makes our dataset skewed, accuracy is not a good measurement for the interaction site prediction. That is why both methods seemed to have high accuracy (94.93 vs. 96.97 %), but the ipHMM model did not perform very well on the interacting residues (with lower recall and precision) in fact.

**Table 2:** Interaction site prediction performance of different models

	Avg. accuracy (%)	Avg. F1 (%)	Avg. MCC (%)	Avg. precision (%)	Avg. recall (%)
ipHMM	94.93	75.61	73.69	77.56	76.51
CM-ipHMM	96.97	90.05	89.11	85.98	96.83
CM-only	96.30	88.52	87.23	85.22	94.91
Ground-truth-CM	99.83	99.51	99.40	99.89	99.21

*ipHMM* the interaction profile hidden Markov model used to prediction interaction site, *CM-ipHMM* the logistic regression model built with the integration of contact matrix prediction and ipHMM interaction site prediction, *CM-only* the logistic regression model built with the predicted contact matrix prediction only, *Ground-truth-CM* the logistic regression model built with the ground-truth contact matrix prediction and ipHMM interaction site prediction

In order to make sure the improvement is not caused by outliers or noise, we performed a paired *t* test between the MCC scores of the ipHMM model and the integrated CM-ipHMM model. The statistical test showed that the CM-ipHMM model significantly outperforms the ipHMM model with a *p* value, 4.36E−77. This indicates that the imperfect contact matrix site prediction could help the interaction site prediction, which encourages us to work on the improvement of contact matrix predictions and PPIS predictions mutually. Because the CM-ipHMM model used both predicted RR contact matrix and predicted site with ipHMM, we cannot rule out the possibility that the improvement contribution purely comes from the predicted contact matrix. Thus, we designed a test with only the predicted contact matrix as features to the machine learning model. The result is shown with the CM-only row in Table 2. We can see the CM-only model also have significant improvement over the ipHMM model. From the MCC scores, we can see that the CM-only model (87.23 %) is closer to the CM-ipHMM model (89.11 %) than the ipHMM model (73.69 %). This implies that the predict contact matrix contributes more than the predicted interaction sites with ipHMM to the integrated CM-ipHMM model. However, this does not mean that we can ignore the contribution of the predicted interaction sites with the ipHMM model. We performed a paired *t* test between the MCC scores of the CM-only model and the integrated CM-ipHMM model. The statistical test showed that the CM-ipHMM model significantly outperforms the CM-only model with a *p* value, 9.32E−10. The ground-truth CM row in Table 2 shows the prediction result if we replace the predicted contact matrix with ground-truth contact matrix. The measurements are close to 100 %. That implies that if we have a perfect prediction on the contact matrix, we could get an almost perfect prediction on the interaction site prediction. This indicates that the upper bound of our CM-ipHMM model could have an almost perfect prediction as we expected. It is not 100 % correct because it is based on the logistic regression model which treated the rows/columns of the predicted contact matrix as features. There are training errors introduced during the training of the CM-ipHMM model.

The weights of the CM-ipHMM model with logistic regression could show the relative importance of each input features. We found that the last feature, which is the ipHMM site prediction feature, has a large positive weight for most cases. That indicates that the ipHMM site prediction result is a significant predictor for our CM-ipHMM model. We used L2 penalty for the logistic regression mode to prevent overfitting during training process, besides the standard cross-validation that uses the test data not seen during the training. Additionally, we also monitor the performance gap between using the predicted contact matrix as features and using the ground-truth contact matrix as features to feed into the logistical regression classifier; the observed gap (e.g., average precision is roughly 85 % for using predicted contact matrix versus 99 % for using the ground-truth contact matrix, in Table 2) does not seem to suggest any overfitting.

In this research, we used a logistic regression model as the classifier for the CM-ipHMM model. However, we are not limited to this classifier. Other machine learning models (such as SVM, Random Forest, Deep Neural Networks, and so on) could be used to replace the logistic regression for the CM-ipHMM model in the future. Also, we used the method in Gonzalex et al. [11] to predict contact matrix, while in principle, this can be done by any method that is designed to make such prediction, such as Ovchinnikov et al. [12]. Furthermore, in addition to the contact matrix prediction features and the ipHMM prediction feature, we could add other helpful features to the machine learning model in the future. For example, conservation scores, sequence homology, physicochemical characteristics, and propensity were found to be helpful for interaction site prediction in the literature [3]. Note that although this work is about the contact points and interaction sites on the interface between interacting proteins, the method can be potentially applicable to similar tasks in protein folding and can benefit from methods developed therein [30–32].

To give a sense of how our method would compare with other interaction site prediction methods, we list the performance of some existing machine learning based methods. Šikić et al. [33] reported a sliding window approach combined with the Random Forests method to predict protein interaction sites. The prediction performance of this method with a combination of sequence and structure-derived parameters reached a precision of 76 % and a recall of 38 % when combined with structural information. Zhou and Shan [2] reported a neural network method with sequence profiles of neighboring residues and solvent exposure as input to predict protein-protein interaction sites. This method achieved a precision of 70 % and a recall of 65 % with non-homologous complex-forming proteins. Yan et al. [34] reported a sequence-based prediction of protein-protein interaction sites approach with SVM. This approach reached a recall of 82.3 % and a specificity of 81.0 % for proteins in the antigen-antibody complexes. Because all these methods used different data sources, it is not straightforward to make a completely fair comparison. However, we can see that the precision of 77.56 % for the ipHMM method is at a similar level with those reported methods and that our CM-ipHMM method significantly outperforms the others per their individually reported performance. Even though whichever method may turn out to be truly the single best performer can only be determined by a head-to-head comparison on a common dataset, our work clearly demonstrates a novel way to extract useful features and a unique way of integrating contact matrix prediction and interaction sites prediction to enhance the latter.

## Conclusions

A novel method, CM-ipHMM, was proposed for the protein interaction site prediction with the integration of contact matrix prediction and ipHMM interaction site prediction. The CM-ipHMM model showed significant improvement over the previous model using ipHMM interaction site prediction only. It demonstrates that the downstream contact matrix prediction can help the interaction site prediction in return, which encourages us to work on the improvement of those predictions mutually. Although the predicted contact matrix model contributes more to the CM-ipHMM model, the predicted interaction site with ipHMM is still an important feature for the CM-ipHMM model. If we can have a good prediction on the contact matrix prediction, the CM-ipHMM model could generate good results for the protein interaction site prediction.
